# Mesoscopic Quantum Effect: The Interaction of Electron Phenomena at the Mesoscopic Scale

**DOI:** 10.3390/nano16010028

**Published:** 2025-12-24

**Authors:** Kai Chen, Laijun Liu

**Affiliations:** 1 School of Physics, Nanjing University of Science and Technology, Nanjing 210094, China; 2 College of Materials Science and Engineering, Guilin University of Technology, Guilin 541004, China; ljliu@163.com

Nanomaterials are, in essence, the many-body system, because of their interactions, correlations, and couplings in condensed matters. In the nanomaterials, a specific quantity of atoms is integrated into the entity at the mesoscopic scale, which is not the case with bulk, film and two-dimensional materials. The interacting electrons even appear to compete for the emergent phenomena as a transition zone is formed between the macroscopic quantum effect and the microscopic quantum effect. In line with classic physics, these phenomena are classified as the mechanic, the thermal, the optical, the electrical, and the magnetic.

Strain is a response to stress at the macroscopic scale. In a classic dielectric physics model, it couples with the ionic off-center displacement, while the displacement generates the dipole moment as an atomic-scale single-body approximation of electric polarization. Naturally, in BaTiO_3_ perovskite, this causes a piezoelectric effect [1]. To enhance this, the dopant of heterogeneous atoms is expected to modify the displacement, especially in the perovskite-based family of materials. This goal is achieved by doping the Li/Sm atoms in the bismuth layer-structured ferroelectric Na_0.5_Bi_2.5_Nb_2_O_9_, which consists of pseudo-perovskite [(Na_0.5_Bi_0.5_)Nb_2_O_7_]^2−^ layers interleaved with (Bi_2_O_2_)^2+^ layers along the *c*-axis, in the (Bi_2_O_2_)^2+^(A_m−1_B_m_O_3_*_m_*_+1_)^2−^ structural model [2]. When the piezoelectric coefficient *d_33_* is increased up to 30.3 pC/N by doping the (LiCe_0.5_Nd_0.5_) atoms, the multiscale effect includes that, the reduced domain walls become more sensitive to the stress with the width of strip-like ferroelectric domains being decreased from 66.57 to 64.75 nm [3]. However, the mechanism is inconsistent with the real-world ceramic conditions. In the Pb_0.92_Ba_0.08_[Zr_0.50+*x*_Ti_0.48−*x*_(Nb_0.5_Sb_0.5_)_0.02_]O_3_ ceramic material, the piezoelectric coefficient *d*_33_ is 855 pC/N and the piezoelectric strain *d*_33_ * is 860 pm/V [4], revealing that ceramic pellets with a thickness of 1.4 mm may shrink or expand at the electron scale, because the effective radius of an electron is about 2.82 pm, and, notably, the ionic lattice is bounded by interacting electrons. In fact, the stress may deform the electron clouds rather than may displace the ions in such hard and brittle crystalline solids. Due to the nature of electric polarization, the piezoelectric effect exhibits a kind of macroscopic quantum effect.

Thermodynamics is a key to the synthesis of nanomaterials. The in situ thermal (IST) method is employed to synthesize the zeolite–imidazole-framework nanocomposites, i.e., Fe-ZIF-8 and Fe-ZIF-67 [5]. Significantly, the adsorption capacities and the removal efficiencies are increased due to the π–π stacking interactions, the hydrogen bonding, and the electrostatic attraction. These nanomaterials’ exceptional efficacy, favorable stability, and substantial specific surface area, demonstrate their potential for eliminating the malachite green and other cationic organic dyes from the aqueous environments. Additionally, the MnSe_2_/CNT nanocomposites, synthesized by a straightforward hydrothermal method, can function as the cathodes of aqueous copper ion batteries, exhibiting a high discharge specific capacity of 545 mAh·g^−1^ after cycling 1000 times at a current density of 2 A·g^−1^ [6]. In addition, the *pH* control cannot be ignored in thermodynamics process. An initial solution with a *pH* value of 12 is used to synthesize Ag@CeO_2_ nanoparticles [7], enhancing their antioxidant and antitumor properties to be more suitable for the treatment of tumors. Thermal methods are also effective for the synthesis of biological nanomaterials. Blood cell membrane-coated nanomaterials are summarized in a review work to demonstrate the recent developments in photodynamic and photothermal treatments for antitumor therapy [8]. Theoretical simulation can provide the valuable reference data for the experiments. For the graphene with lithium-modified groups, the feasibility of thermodynamic methods is assessed through the simulation, aiming to enhance the detection of hydrogen [9]. To analyze temperature distribution across a Ti-6Al-4V titanium alloy sheet, a coupled electro-thermal-mechanical analysis is simplified by a preheating flux model in a coupled thermal–mechanical simulation. Consequently, a design criterion for electrode length in integral electric hot incremental forming is obtained [10]. In another study, the effects of size and ratio are simulated for Ru–Zn nanoparticles to elucidate the role played by interacting electrons in the excellent catalytic performance of these materials, especially regarding the selective hydrogenation of benzene to cyclohexene in the industrial production [11]. To obtain a high-energy-density polymer with robust thermodynamic stability under high pressures, a method of extracting polymeric nitrogen N_10_ from the host–guest ArN_10_ compound by using ab initio calculation is proposed. Notably, N_10_ with an energy density twice that of TNT is obtained [12].

Quantized photons, electrons, and phonons, can be described by a unified theory, using a fully atomistic ab initio approach, when they are beyond equilibrium dynamics [13]. This understanding has resulted in the optoelectronic synaptic devices for the in-sensor neuromorphic memory applications [14], the perovskite quantum dot-based memory technologies [15], the large-aperture all-Si metalens-based compact near-infrared imaging devices [16], the piezoelectric/ferroelectric effect-based self-powered deep-ultraviolet photodetectors [17], and the PCBM nanocrystal-incorporated donor–acceptor polymer ultraviolet phototransistors [18]. As a typical example, the nanometer-resolution in situ imaging of thick frozen bio-samples and microchips can achieved by optimizing the electron beam energy of an MeV-STEM [19] and developing the method based on the detailed knowledge of beam emittance, aberrations in the STEM column optics, and the energy-dependent elastic and inelastic critical angles of the materials [20]. As the editors of this collection, we believe that this work represents a milestone in the in situ MeV-STEM imaging technology.

The interactions of electrons are important when it comes to the electrical properties of nanomaterials and the resultant devices. Even at the mesoscopic scale, they may retain their quantum nature and couple with other quantum characteristics. In the *T_d_*-MoTe_2_ -bilayer dielectric nanocomposite of a pseudo-bilayer quantum Hall system, the large polarons condense and distort the local lattice for the polaron-type polarization [21]. During the process of switching polarization, they are paired with the holes to generate a superconducting current. At the macroscopic scale, the reverse ferroelectricity and the superconductivity are coupled. As the editors, we believe that this work suggests the coupling possibility of two or more macroscopic quantum effects. In the multiferroic bismuth ferrite, the interacting electrons form the quasipolarons to condense into the subsurface nanolayer. Because of their quantum nature, they demonstrate the step-like characteristics of a pressure-dependent transient frequency, when they are coupled with the bulk dipole chains [22]. Moreover, the theoretical simulation is beneficial for studying the properties of various nanodevices, including the electronic transport process through T-shaped double quantum dots [23] and the sequence of charge emissions from a floating storage node through a transistor in a subthreshold bias condition [24].

Spin is the microscopic base of magnetism at the macroscopic scale. In a molecular device with a dynamic covalent chemical bridge connected to zigzag graphene nanoribbon electrodes, the spin-dependent transport properties are investigated by combining the density functional theory with the non-equilibrium Green function method [25]. Both excellent properties and novel effects are predicted to be deployed for energy-efficient spintronic logic gates and non-volatile memory devices in the future spintronic applications.

In summary, all of these studies examine how the quantitative change leads to the qualitative change as a result of the many-body interacting electrons. Ultimately, these works prove that there is a mesoscopic quantum effect.
